# Persistent inflammatory activation in people living with HIV. Involvement in atherosclerosis

**DOI:** 10.3389/fmed.2025.1621765

**Published:** 2025-07-11

**Authors:** Francisco Illanes-Álvarez, Denisse Márquez-Ruiz, Sara Cuesta-Sancho, Irene Campaña-Gómez, Andrés Martín-Aspas, Ismael Tinoco-Racero, Mercedes Márquez-Coello, José-Antonio Girón-González

**Affiliations:** ^1^Servicio de Medicina Interna, Hospital Universitario Puerta del Mar, Cádiz, Spain; ^2^Instituto para la Investigación e Innovación Biomédica de Cádiz (INIBiCA), Cádiz, Spain; ^3^Facultad de Medicina, Universidad de Cádiz, Cádiz, Spain; ^4^Mucosal Immunology Lab, Institute of Biomedicine and Molecular Genetics (IBGM, University of Valladolid-CSIC), Valladolid, Spain

**Keywords:** HIV, proinflammatory cytokines, endothelial activation, platelet activation, atherosclerosis, cardiovascular risk factors

## Abstract

**Background:**

An increased prevalence of atherosclerosis has been observed in people living with HIV (PLWH). This study aimed to analyze levels of inflammatory, immune, endothelial, platelet, and coagulation parameters, as well as their relationship with subclinical atherosclerosis.

**Methods:**

A total of 120 PLWH with chronic infection and undetectable HIV load, along with 30 age- and sex-matched controls (HIV-uninfected individuals), were selected. Serum levels of proinflammatory molecules, including interleukin 6 (IL-6), soluble CD163, and high-sensitivity C-reactive protein, were measured. Additionally, neutrophil extracellular traps (NET)-derived parameters (anti-nucleosome antibody titers and myeloperoxidase concentrations), activated T lymphocytes, endothelial (E-selectin, vascular cell adhesion molecule 1), platelet (P-selectin, Platelet factor 4) and coagulation (D-dimer) markers were assessed. Cardiovascular risk factors were evaluated using the REGICOR and ASCVD risk estimators. In a subset of 61 individuals (18 controls and 43 PLWH), subclinical atherosclerosis was assessed by carotid Doppler ultrasound.

**Results:**

Levels of IL-6, sCD163, anti-nucleosome antibodies, and activated T lymphocytes were elevated in PLWH compared to controls. Likewise, serum levels of platelet factor 4 and D-dimer were higher in PLWH. Those PLWH with carotid atherosclerotic lesions exhibited higher REGICOR and ASCVD scores, as well as elevated IL-6 concentrations, compared to those PLWH without carotid atherosclerotic lesions. Multivariable analysis identified the REGICOR score and IL-6 serum levels as independent factors associated with atherosclerosis.

**Conclusion:**

People living with HIV with long-term viral load control exhibit increased levels of inflammatory, immune, platelet and coagulation markers. Subclinical atherosclerosis in this population is independently influenced by both classic cardiovascular risk factors and inflammatory activation.

## Introduction

An increased prevalence of cardiovascular diseases has been observed in people living with HIV (PLWH) compared to the general population, even in the era of effective antiretroviral therapy (ART) (1). As life expectancy in PLWH continues to rise due to sustained viral suppression and immune recovery, non-AIDS comorbidities, particularly cardiovascular diseases, have become a major cause of morbidity and mortality in this population. A better understanding of the mechanisms underlying vascular alterations in PLWH is critical, as this could expand therapeutic possibilities for these patients.

Multiple pathophysiological processes have been proposed to explain the increased cardiovascular risk in PLWH. Among them, endothelial dysfunction, platelet activation, and alterations in coagulation/fibrinolysis have been proposed as mechanisms contributing to this elevated risk (2). In this context, biomarkers such as high-sensitivity C-reactive protein (hsCRP), interleukin-6 (IL-6), and D-dimer have been widely used in large cohort studies and consistently associated with increased cardiovascular risk and mortality in PLWH (3). However, the behavior of these biomarkers in the specific setting of long-term viral suppression and immune restoration remains incompletely understood (4). In fact, findings from various studies have been inconsistent, potentially reflecting heterogeneity in study populations, including differences in ART (antiretroviral therapy) status, levels of viral replication, and degrees of immunosuppression (2, 5, 6). Furthermore, the potential interplay between these established inflammatory markers and emerging contributors —such as neutrophil extracellular traps (NETs)— has not yet been systematically explored.

Chronic immune activation and persistent systemic inflammation are additional mechanisms that may significantly contribute to the elevated prevalence and pathogenesis of cardiovascular disease in PLWH. This persistent immune activation is observed in PLWH even when viral loads are undetectable and current CD4+ T-cell counts are high (1). Elevated levels of inflammatory and immune activation markers have also been associated with increased mortality in this population (7).

Most research has focused on T lymphocytes and macrophages (8), while the role of polymorphonuclear neutrophils (PMNs) and their contribution through processes such as NETosis (formation of NETs, Neutrophil Extracellular Traps) (9, 10) has been underexplored. NETs are structures composed of DNA and proteins that can trap pathogens, but they have also been implicated in sterile inflammation and tissue damage. Notably, NETs have been associated with atherosclerotic plaque formation in the general population (11–13), and elevated NETs levels have been reported in PLWH (14), suggesting that neutrophil activation and NET formation may contribute to HIV-associated vascular disease and warrant further investigation.

In this context, this study had a twofold objective: first, to evaluate the possible presence of inflammatory –including that related to PMNs–, immune, endothelial, platelet, and coagulation activation in a homogenous cohort of PLWH with prolonged viral suppression and elevated CD4+ T cell/mm^3^ counts; second, to explore the association of these biological processes —alongside classical vascular risk factors (smoking, diabetes mellitus, arterial hypertension, hypercholesterolemia)— with subclinical atherosclerosis. By integrating these perspectives, we aim to advance a more comprehensive framework for understanding the multifactorial contributors to atherosclerosis in virologically suppressed PLWH, which could enhance cardiovascular risk stratification and guide targeted therapeutic interventions.

## Materials and methods

### Study design

This prospective observational study was conducted at the HIV outpatient clinics of Puerta del Mar University Hospital (Cádiz, Spain) between 2018 and 2023. We enrolled consecutive people living with HIV (PLWH) who attended routine follow-up visits during this period.

Eligible PLWH were adults (≥ 18 years) with chronic HIV infection who had maintained an undetectable plasma HIV viral load (< 50 copies/mL) for at least 12 months prior to inclusion, on stable antiretroviral therapy (ART). Patients with any active opportunistic infection, concomitant neoplasm, or other inflammatory conditions were excluded. Additional exclusion criteria included active substance abuse (cocaine, heroin, amphetamines), significant alcohol consumption (> 50 g/day), recent blood transfusions (within 30 days), or use of anti-inflammatory or immunosuppressive medications that could affect inflammatory markers.

The screening procedures for PLWH followed established national protocols (15).

A total of 120 PLWH meeting these criteria were included. Their ART regimens consisted of tenofovir alafenamide plus emtricitabine (64 patients, 53%) or abacavir plus lamivudine (56 patients, 47%), combined with rilpivirine (22 patients, 18%), integrase inhibitors (31 patients, 56%), or the protease inhibitor darunavir (31 patients, 26%).

The control group comprised 30 age- and sex-matched healthy individuals recruited from hospital staff and community volunteers during the same period. Controls were screened to exclude any history of HIV infection, cardiovascular disease, chronic inflammatory conditions, or use of medications affecting immune function.

All participants (PLWH and controls) were asymptomatic for cardiovascular or other target organ diseases (ischemic heart disease, cerebrovascular disease or lower extremity atherosclerosis) at inclusion.

### Definitions

The duration of the HIV infection was established based on the first positive anti-HIV test. HIV replication was considered controlled when the viral load was below 50 copies/ml (Abbott RealTime HIV-1, Abbott Park, IL, United States).

Increased serum concentration of intestinal fatty acid-binding protein (I-FABP) was indicative of gut barrier disruption (16). Bacterial translocation was assessed by measuring plasma 16S ribosomal RNA (16S rRNA) levels (17).

Markers of inflammatory activation included serum concentrations of interleukin (IL)-6, soluble CD163 (sCD163) and high sensitivity C-reactive protein (hsCRP). Serum titers of anti-nucleosome IgG antibodies (AnuA-IgG) and serum levels of myeloperoxidase (MPO) were indicative of NET formation. Serum levels of DNAse I were analyzed as the main enzyme responsible for NET degradation (18). Activated CD4+ and CD8+ T lymphocytes were identified by the co-expression of HLA-DR and CD38 on their membranes. Markers of vascular activation included serum concentrations of vascular cell adhesion molecule 1 (VCAM-1) and E-selectin. Platelet activation was assessed through levels of P-selectin and chemokine (C-X-C motif) ligand 4/Platelet factor 4 (CXCL4/PF4). D-dimer concentration was used to assess coagulation/fibrinolysis.

Participants who smoked more than 10 cigarettes per day were considered smokers. Diagnostic criteria for dyslipidemia, diabetes mellitus, arterial hypertension, and cardiovascular or cerebrovascular disease followed the latest clinical guidelines from the Spanish Society of Atherosclerosis (19). Patients were categorized using two vascular risk assessment tools: the Framingham scale adapted for the Spanish population (REGICOR) (20) and the American College of Cardiology’s cardiovascular risk scale (ASCVD risk estimator) (21). The REGICOR scale includes age, sex, smoking status, diagnosis of diabetes mellitus, serum levels of total cholesterol and high-density lipoprotein cholesterol, as well as systolic and diastolic arterial pressure. The ASCVD risk estimator additionally considers race, low-density lipoprotein cholesterol, and the use of antihypertensive medications, statins or aspirin, and has been recommended for use in patients with HIV infection.

### Study schedule

The study protocol included: (1) collection of clinical history, nadir CD4^+^ T cell count, and duration of undetectable HIV load; (2) CD4^+^ T cell count at study inclusion; (3) peripheral blood sampling for analysis of bacterial translocation, inflammatory and immune parameters, as well as vascular and platelet activation and coagulation/fibrinolysis markers; (4) peripheral blood sampling for measurement of cholesterol, LDL-cholesterol, HDL-cholesterol, triglyceride concentrations, total cholesterol/HDL ratio, and glycemia; (5) in a subgroup of consecutive individuals (18 controls and 43 PLWH), carotid Doppler ultrasound was performed.

A comparison of the results for activated lymphocytes and parameters of inflammatory, vascular, and platelet activation, as well as ultrasound-Doppler findings, was conducted between controls and PLWH.

### 16S rDNA levels analysis

16S rDNA was extracted using the QIAamp DNA Blood Mini Kit (QIAgen, Hilden, Germany) with modifications to the manufacturer’s protocol. Briefly, plasma was obtained by centrifuging blood samples collected in ethylenediaminetetraacetic acid (EDTA) tubes at 2,500 g for 15 min at room temperature. The plasma was then frozen at −80°C until use. For extraction, 1 ml plasma was mixed with 15 μl of proteinase K and 1 ml of buffer AL, followed by a 30 min incubation at 56°C. Subsequently, 1 ml of 100% ethanol was added, and the mixture was incubated at room temperature for 10 min. The content was then processed through a spin column in multiple centrifugation steps. Washing steps were performed using 1 ml of buffer AW1 and 1 ml of buffer AW2, with elution carried out using 25 μl of buffer AE. The eluate underwent a 5 min incubation at 56°C, followed by centrifugation at 10,000 rpm for 30 s, re-collection, and a final centrifugation at 10,000 rpm for 1 min.

The 16S rDNA region of *Escherichia coli* was amplified in a CFX Connect Real-Time PCR System (CFX Connect Real-Time PCR System, Bio-Rad Laboratories, Inc. CA, United States) using the following primers: 16S F 5′-AGA-GTT-TGA-TCA-TGG-CTC-AG-3′ and 16S R 5′-ACC-GCC-ACT-GCT-GCT-GGC-AC-3′ (IDT, Coralville, Iowa, United States) (22). A standard curve was generated from an *E. coli* colony, provided by the Microbiology Department of Puerta del Mar University Hospital. After heat shock (90°C for 10 min and 10°C for 10 min) and centrifugation, the supernatant was collected and the concentration was measured with a Qubit fluorimeter and the Qubit™ dsDNA HS Assay Kit (Invitrogen, Thermo Scientific, Waltham, MA, Uinited States). Samples were run in duplicates, and their concentrations were determined using the standard curve generated from serial dilutions of the colony. All procedures were performed in a biological safety horizontal flow hood.

### Microbial translocation markers, proinflammatory molecules, DNAse I, vascular- and platelet-related parameters, and D-dimer concentration

Serum was obtained by centrifuging blood samples collected in pyrogen-free heparinized tubes (Biofreeze, Costar, United States) at 2,500 g for 15 min at room temperature, with centrifugation performed immediately after blood extraction, in order to minimize potential changes in cytokine concentrations. The serum was then frozen at −80°C until its use.

Quantikine Human Immunoassays (R&D, Minneapolis, MN, United States) were used to quantify serum concentrations of I-FABP, IL-6, sCD163, hsCRP, MPO, VCAM-1, E-selectin, P-selectin, and PF4, following the indications of the manufacturer. Serum concentrations of anti-nucleosome IgG antibodies were measured using the Human anti-nucleosome antibody IgG (AnuA-IgG) ELISA Kit (MyBioSource, San Diego, CA, United States). DNAse I concentrations were determined with the Human DNase-I (deoxyribonuclease I) ELISA Kit (Biomatik, Wilmington, DE, United States). Plasma D-dimer concentration was analyzed by immunoassay using the D-Dimer HS 500 kit (Instrumentation Laboratory, Bedford, MA, United States). The samples were run in duplicate.

### T-Cell immune phenotypes

Fresh blood samples collected in pyrogen-free heparinized tubes with EDTA (Biofreeze, Costar, United States) were used for flow cytometry. Stained cells were acquired on a BD FACSCanto™ II flow cytometer using BD FACSDiva™ Software (BD Biosciences, San Jose, CA, United States), and the resulting data were subsequently analyzed with FlowJo™ Software (BD Biosciences, San Jose, CA, United States). Activated CD4+ and CD8+ T lymphocytes (HLA-DR+CD38+) were identified using a multi-step gating strategy. Initially, viable cells were identified using the FVS510 viability marker (BD Biosciences, San Jose, CA, United States). From this population, lymphocytes were selected based on their characteristic forward (FSC-A) and side (SSC-A) scatter. Then, the CD3+ population was selected (clone SK7, BD Biosciences, San Jose, CA, United States), and it was further divided into CD4+ (clone SK3, BD Biosciences, San Jose, CA, United States) and CD8+ (clone SK1, BD Biosciences, San Jose, CA, United States) populations. Finally, activated T cells were defined within each CD4+ and CD8+ subset by the co-expression of activation markers CD38+ (clone HB-7, BD Biosciences, San Jose, CA, United States) and HLA-DR+ (clone L243, BD Biosciences, San Jose, CA, United States) (Supplementary Figure 1). In each case, 300,000 cells were acquired. Fluorescence minus one (FMO) controls were used to confirm staining specificity and distinguish the sample from background.

### Evaluation of the carotid artery

Patients and controls underwent ultrasound measurements of carotid intima-media thickness (CIMT) using a Toshiba F31 device, equipped with a linear probe UST-5413 (4–11 MHz). Individuals were examined in the supine position, with their head turned 45° away from the side being studied. Three carotid segments were studied: the common carotid artery (1 cm proximal to bifurcation), the carotid bulb, and the internal carotid artery (1 cm distal to bifurcation). Using computer software with automatic edge detection integrated in the device, the maximum and mean values of the CIMT were measured for each segment, considering the average intimate-media thickness across all zones. The atheromatous plaque was defined as: (a) Focal thickening of the wall at least 50% higher than the surrounding wall. (b) Focal thickening of the wall that penetrates 0.5 mm in the lumen. (c) Localized area with a CIMT of more than 1.5 mm that penetrates the lumen and that is identified differentiated from the surrounding area. Carotid lesion was defined as a CIMT value exceeding 0.9 mm and/or the presence of plaques (23). The cut-off point set in the present study (0.9 mm) follows the consensus of the American Society of Echocardiography, which suggests that CIMT values at or above the 75th percentile of a reference population indicate increased cardiovascular risk (24); this value was 0.88 mm in a sample of the Spanish reference population (25).

### Statistics

Data were expressed as absolute numbers (percentage) or as median values [25–75 interquartile range (IQR)]. Categorical variables were compared using the chi-square test or Fisher’s exact test. The Mann-Whitney U test was used to compare quantitative variables from two independent groups. For comparison of three or more independent groups, Kruskal-Wallis test was used. Spearman rank correlation tests were performed to evaluate associations between two variables. A two-tailed *p*-value of < 0.05 was considered to be significant.

Multivariable analysis of factors associated with the presence of carotid lesion was performed using binary logistic regression. A stepwise backward elimination approach was applied, starting with all candidate variables and sequentially removing those with the least statistical significance. The independent variables included were age, sex, duration of undetectable HIV load, and those selected from bivariate regression analysis using a cutoff *p-*value of < 0̃.1.

SPSS 22.0 statistical software package (SPSS Inc., Chicago, IL, United States) was used to perform these analyses.

## Results

The demographic and immune characteristics, as well as the cardiovascular indexes of PLWH and controls, are shown in Table 1.

**TABLE 1:** Demographic, clinical, immunological, and cardiovascular risk characteristics of controls and people living with HIV.

Parameter	Controls (*n* = 30)	People living with HIV (*n* = 120)	*P*
Age (years)	53 (37–58)	54 (42–59)	0.256
Sex male (*n*, %)	22 (73)	94 (78)	0.633
MSM as risk factor for HIV infection (*n*, %)	−	63 (53)	−
Time of evolution of HIV infection (months)	−	132 (36–188)	−
**Immune-virological variables**			
CD4+ T cell/mm^3^ at diagnosis	−	362 (177–600)	−
CD4+ T cell/mm^3^ at inclusion	1,235 (866–1723)	677 (526–969)	**< 0.001**
CD8+ T cell/mm^3^ at inclusion	350 (254–588)	933 (694–1,383)	**< 0.001**
Time with undetectable HIV load (months)	−	86 (13–144)	−
**Risk factors for atherosclerosis and target organ impairment**			
Diabetes mellitus (*n*, %)	1 (3)	13 (11)	0.302
Hypercholesterolemia (*n*, %)	5 (17)	55 (46)	**0.003**
Smokers (*n*, %)	0 (0)	38 (31)	**< 0.001**
Arterial hypertension (*n*, %)	0 (0)	28 (23)	**0.001**
Cardiovascular risk (%) at 10 years, according to the scale			
REGICOR	1 (1–1)	3 (2–5)	**< 0.001**
ASCVD	0 (0–0)	6 (3–11)	**< 0.001**
Carotid intima-media thickness (mm)*	0.73 (0.70–0.80)	0.75 (0.68–0.91)	0.486
At least one atherosclerotic plaque (*n*, %)*	4 (22)	20 (47)	0.092
Target carotid damage according ultrasound (*n*, %)*	5 (28)	21 (49)	0.163

*Carotid Doppler ultrasound was performed in 61 individuals (18 controls and 43 patients living with HIV). Data are presented as absolute number (percentage) for categorical variables and as median (interquartile range) for continuous variables. Comparisons between groups were performed using the Mann-Whitney U test for continuous variables and the chi-square or Fisher’s exact test for categorical variables. Statistically significant *p*-values (< 0.05) are shown in bold. ASCVD, American College of Cardiology/American Heart Association atherosclerotic cardiovascular disease risk score. MSM, men who have sex with men. REGICOR, cardiovascular risk score adapted from the Framingham scale for the Spanish population.

### Intestinal barrier permeability and bacterial translocation

Intestinal permeability (measured by serum I-FABP concentration) and bacterial translocation (measured by 16S rDNA values) were significantly higher in PLWH compared to controls (Table 2).

**TABLE 2:** Gut barrier integrity, bacterial translocation, and inflammatory, immune, vascular, platelet, and coagulation parameters in controls and chronically treated people living with HIV.

Parameter	Controls (*n* = 30)	People living with HIV (*n* = 120)	*P*
Intestinal fatty acid binding protein (pg/ml)	1,519 (1,114–2,207)	2,304 (1,466–3,489)	**0.001**
16S rDNA (ng/ml)	9.8 (5.8–13.0)	12.2 (8.2–26.3)	**0.049**
IL-6 (pg/ml)	2.0 (1.5–2.5)	3.6 (2.1–4.9)	**< 0.001**
sCD163 (ng/ml)	536 (437–620)	686 (502–776)	**0.030**
High sensitivity C reactive protein (ng/ml)	2,756 (1,569–6,337)	5,635 (3,392–7,782)	0.061
CD4+ T cell/mm^3^	1,256 (861–1,788)	731 (533–959)	**< 0.001**
CD4+CD38+DR+ (percentage of CD4+ T lymphocytes)	0.3 (0.2–0.9)	1.9 (1.0–4.0)	**< 0.001**
CD8+ T cells/mm^3^	350 (254–588)	955 (696–1,385)	**< 0.001**
CD8+CD38+DR+ (percentage of CD8+ T lymphocytes)	0.8 (0.4–1.6)	3.0 (1.4–9.0)	**< 0.001**
PMNs (cells/mm^3^)	3,370 (2,585–3,865)	3,530 (2,850–4,540)	0.262
Anti-nucleosomes IgG (pg/ml)	1,539 (877–2,330)	2,025 (1,247–4,428)	**0.036**
Myeloperoxidase (ng/ml)	243 (89–406)	300 (212–493)	0.329
DNAse I (ng/ml)	9 (5–15)	15 (7–23)	**0.005**
E-selectin (ng/ml)	36 (30–47)	42 (30–52)	0.205
VCAM-1 (ng/ml)	678 (541–879)	606 (455–873)	0.395
Platelets (1,000 × cells/mm^3^)	226 (191–263)	226 (192–258)	0.943
P-selectin (ng/ml)	112 (69–150)	115 (70–163)	0.564
PF4 (μg/ml)	13 (12–14)	15 (13–16)	**0.002**
D-dimer (μg/ml)	280 (177–380)	417 (325–518)	**0.012**

Data are presented as median (interquartile range). Comparisons between groups were performed using the Mann-Whitney U test. IgG, immunoglobulin G. IL-6, interleukin 6; sCD163, soluble CD163; PMNs, polymorphonuclear neutrophils; NET, neutrophil extracellular traps; PF4, platelet factor 4 (chemokine (C-X-C motif) ligand 4); VCAM-1, vascular cell adhesion molecule 1. Statistically significant *p*-values (< 0.05) are shown in bold.

### Inflammatory and immune activation parameters

Serum concentrations of inflammatory activation markers (IL-6, sCD163 and AnuA-IgG) were elevated in PLWH, with hsCRP levels also approaching statistical significance. The concentrations of DNAse I were likewise higher in this group, while serum MPO levels were similar between PLWH and controls (Table 2). Furthermore, the proportion of activated CD4+ and CD8+ T cells was significantly higher in PLWH (Table 2).

**FIGURE 1:**
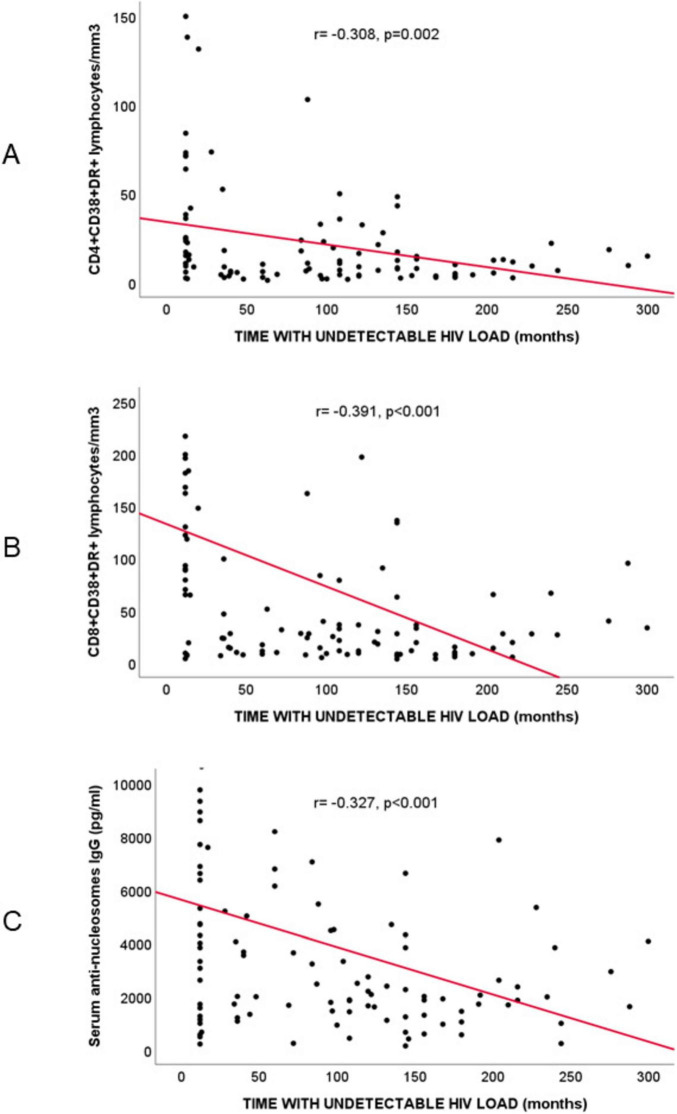
Spearman’s correlation analysis in people living with HIV (*n* = 120) between time with undetectable HIV viral load (months) and: **(A)** activated CD4+ T lymphocytes (CD4+CD38+DR+T lymphocytes/mm^3^; r = –0.308, *p* = 0.002); **(B)** activated CD8+ T lymphocytes (CD8+CD38+DR+T lymphocytes/mm^3^; r = –0.391, *p* < 0.001); **(C)** serum anti-nucleosome IgG titers (pg/ml; r = –0.327, *p* < 0.001).

A significant negative correlation was detected between the duration of undetectable HIV viral load and AnuA-IgG titers (r = −0.327, *p* < 0.001) (Figure 1). When only individuals with more than 18 months of undetectable viral load were analyzed, the negative correlation between duration of viral suppression and AnuA-IgG titers remained significant (r = −0.267, *p* = 0.021). Likewise, the correlation between time with undetectable viral load and anti-nucleosomes IgG concentration persisted when the sample was stratified based on whether the age of PLWH was younger (r = −0.301, *p* = 0.024) or older (r = −0.285, *p* = 0.042) than their median age (54 years). These findings highlight the robustness of the association between reduced AnuA-IgG titers and prolonged viral suppression.

### Vascular and platelet activation markers and D-dimer levels

No significant differences were observed in serum E-selectin or VCAM-1 concentrations between PWLH and controls. Likewise, serum concentration of P-selectin was similar in both groups; however, a significant increase of PF4 was detected in PLWH. D-dimer concentration was also significantly higher in PLWH compared to controls (Table 2).

### Atherosclerosis-related measures. Relationship with other inflammatory parameters

The 10 years cardiovascular risk, calculated using both the REGICOR and the ASCVD scales prior to carotid Doppler ultrasound, was significantly higher in PLWH.

People living with HIV who underwent carotid Doppler ultrasound were divided into two groups based on the presence (*n* = 21) or absence (*n* = 22) of carotid lesions. The 10 years cardiovascular risk was significantly higher in those with carotid lesions than in those without. Furthermore, PLWH with carotid atherosclerotic lesions exhibited significantly higher IL-6 concentrations and PMN/mm^3^ counts compared to those without such lesions (Table 3).

**TABLE 3:** Gut barrier integrity, bacterial translocation and inflammatory, immune, vascular, and platelet parameters in chronically treated people living with HIV, grouped by the presence or absence of carotid lesions.

Parameter	PLWH without carotid defined lesion (*n* = 22)	PLWH with carotid defined lesion (*n* = 21)	*P*
Age (years)	58 (51–61)	59 (55–62)	0.330
Sex male (*n*, %)	15 (68)	14 (67)	1.000
CD4+ T cell/mm^3^ at diagnosis	374 (161–438)	220 (92–355)	0.354
Time with undetectable HIV load (months)	123 (87–212)	144 (103–186)	0.827
Cardiovascular risk (%) at 10 years (calculated prior to knowing the result of the cervical Doppler ultrasound), according to the scale			
REGICOR	3 (2–3)	4 (3–5)	**0.003**
ASCVD	4 (2–8)	8 (4–12)	**0.037**
Intestinal fatty acid binding protein (pg/ml)	2,761 (2,089–3,483)	2,866 (1,561–4,575)	0.900
16S rDNA (ng/ml)	38 (22–72)	108 (21–143)	0.315
Serum IL-6 (pg/ml)	2.2 (1.9–4.1)	3.6 (2.3–6.5)	**0.027**
Serum CD163s (ng/ml)	645 (479–732)	696 (518–794)	0.262
High sensitivity C reactive protein (ng/ml)	4,460 (1,715–7,661)	5,224 (3,013–8,447)	0.570
CD4+ T cells/mm^3^	655 (563–791)	733 (517–998)	0.308
CD4+CD38+DR+ (percentage of CD4+ T lymphocytes)	1.0 (0.5–2.0)	0.7 (0.4–1.2)	0.276
CD8+ T cells/mm^3^	957 (487–1,398)	1,146 (806-1,378)	0.302
CD8+CD38+DR+ (percentage of CD8+ T lymphocytes)	2.0 (0.8–4.0)	1.2 (0.6–1.9)	0.126
PMNs (cells/mm^3^)	3,035 (2,058–3,640)	3,540 (2,920–5,280)	0.040
Anti-nucleosomes IgG (pg/ml)	1,707 (955-2,734)	1,850 (1,339–2288)	0.964
Myeloperoxidase (ng/ml)	261 (161–343)	339 (132–627)	0.461
DNAse I (ng/ml)	15 (5–27)	11 (4-18)	0.365
E-selectin (ng/ml)	46 (36–56)	50 (36–57)	0.562
VCAM-1 (ng/ml)	764 (600–1,023)	904 (640–1,064)	0.512
Platelets (1000 × cells/mm^3^)	228 (177–263)	223 (201–254)	0.971
P-selectin (ng/ml)	124 (64–161)	114 (69–171)	0.688
PF4 (μg/ml)	15 (13–17)	14 (13–15)	0.657
D-dimer (ng/ml)	410 (341–497)	424 (419–486)	0.368
Carotid intima-media thickness (mm)	0.72 (0.68–0.75)	0.91 (0.73–1.08)	**0.002**

Data are presented as absolute number (percentage) for categorical variables and as median (interquartile range) for continuous variables. Comparisons between groups were performed using the Mann-Whitney U test for continuous variables and the chi-square or Fisher’s exact test for categorical variables. Statistically significant *p*-values (< 0.05) are shown in bold. Carotid lesions were defined by Doppler ultrasound criteria. ASCVD, American College of Cardiology/American Heart Association atherosclerotic cardiovascular disease risk score; IgG, immunoglobulin G; IL-6, interleukin 6; sCD163, soluble CD163; PMNs, polymorphonuclear neutrophils; PF4, platelet factor 4 (chemokine (C-X-C motif) ligand 4); REGICOR, cardiovascular risk score adapted from the Framingham scale for the Spanish population; VCAM-1, vascular cell adhesion molecule 1.

Binary logistic regression analysis identified the REGICOR index and serum IL-6 concentration as independent parameters associated with the presence of carotid lesions (Table 4).

**TABLE 4:** Parameters independently associated with subclinical carotid atherosclerosis in people living with HIV (PLWH) (binary logistic regression).

Parameter	Exp (B)	95% confidence interval		*P*
Age (years)	0.936	0.745	1.175	0.568
Sex (male)	1.759	0.335	9.250	0.505
Time with undetectable HIV load (months)	1.003	0.992	1.014	0.612
Cardiovascular risk at 10 years according to the ASCVD scale	1.098	0.853	1.412	0.467
Cardiovascular risk at 10 years according to the REGICOR scale	2.112	1.096	4.070	**0.026**
Interleukin 6 (pg/ml)	1.568	1.038	2.369	**0.033**

ASCVD, American College of Cardiology/American Heart Association atherosclerotic cardiovascular disease risk score; REGICOR, cardiovascular risk score adapted from the Framingham scale for the Spanish population. Statistically significant *p*-values (< 0.05) are shown in bold.

## Discussion

Previous studies have shown that PLWH have a higher prevalence of atherosclerosis (26). To further analyze the possible pathogenic factors contributing to this increased prevalence, the present work evaluated a range of markers —specifically those related to inflammatory, immune, endothelial, platelet, and coagulation activation— and their possible influence on the accelerated development of atherosclerosis in a selected and homogeneous cohort of patients with well-controlled viral replication over an extended period. This population, characterized by high CD4+ T cell counts and a low incidence of opportunistic infections, provides an optimal model for examining non-AIDS comorbidities, with cardiovascular disease emerging as a primary concern (1).

Consistent with other authors (27), our results suggest that increased intestinal permeability and persistent bacterial antigenic stimulation are present in these individuals, even after prolonged control of HIV viral replication. Bacterial translocation contributes to chronic immune activation —a hallmark of HIV infection— and is considered a central driver of persistent inflammation and immune activation in PLWH (27, 28).

Likewise, increased lymphocyte activation and higher concentrations of IL-6 and sCD163 were observed in PLWH, supporting the hypothesis that chronic inflammation is a key mediator of HIV-associated cardiovascular risk (29). Inflammatory markers derived from PMNs have been insufficiently studied in this context. In this study, an indirect measure of NETs was used —specifically, the immune response to nucleosomes released during NETosis (AnuA-IgG titers)—, which was found to be elevated in PLWH, suggesting the presence of circulating nucleosomes derived from NETs. Serum concentrations of DNAse I were also increased in PLWH, probably as a compensatory mechanism to control the excessive NETosis detected in this group.

Previous authors had analyzed NET formation in a heterogeneous sample of PLWH, reporting elevated serum concentration of citrullinated histone H3 (H3Cit), but not cell-free DNA, as indirect measures of NET formation, regardless of HIV viral load status (14). These measurements were also conducted in our work, but the high variability of the results obtained (data not shown) led to their exclusion from the final analysis. Interestingly, serum levels of the other marker of NETs studied, MPO, was similar between PLWH and controls. This finding leads us to hypothesize that MPO, and possibly other enzymes secreted by PMNs, might be trapped within these neutrophilic networks, rendering them less detectable in serum. Together, these findings suggest that NETosis may be active in virologically suppressed PLWH and could contribute to vascular inflammation.

Furthermore, the concentration of anti-nucleosome antibodies was negatively correlated with the duration of undetectable HIV viral load, suggesting that longer periods of viral replication control are associated with reduced activation of PMNs. This correlation persisted even when the analysis was restricted to PLWH with more than 18 months of controlled viral load, supporting the potential protective effect of long-term viral suppression on inflammatory homeostasis.

While endothelial dysfunction and platelet and coagulation activation have been proposed as mechanisms contributing to cardiovascular risk in PLWH (2), our findings challenge their role in patients receiving prolonged ART. HIV infection and endothelial activation can induce the shedding of cellular adhesion molecules such as E-selectin and VCAM-1 (30, 31). Given the persistent inflammatory state observed in these individuals, it would be expected that endothelial activation would also persist. However, no evidence of ongoing endothelial activation, as measured by serum concentrations of adhesion molecules E-selectin and VCAM-1, was found in PLWH. Prospective studies have shown that after 1 year of ART, the concentration of endothelial activation molecules tends to normalize (5), which supports our findings. However, we cannot exclude the possibility of endothelial activation mediated by other markers or pathways not assessed in this work (32).

Similarly, in our work, P-selectin concentrations—an indicator of platelet-endothelial interaction—did not differ between PLWH and controls, consistent with previous reports showing decreased P-selectin levels following 12 months of ART (6). In contrast, the serum concentration of PF4, a parameter not previously analyzed in PLWH, was increased in our cohort. PF4 is the most abundant protein in platelet alpha granules (33) and serves as both a marker of platelet activation and a mediator of tissue fibrosis (32). These findings suggest that, beyond platelet dysfunction, potential tissue fibrosis (arterial or cardiac) may contribute to cardiovascular events in PLWH.

Finally, our results confirmed elevated D-dimer concentrations in PLWH, a marker that has been independently associated with mortality in individuals with controlled HIV replication (3). This finding underscores the persistent hypercoagulable state in virologically suppressed patients and highlights the need for ongoing monitoring of thrombotic risk.

Taken together, our results demonstrate that even after a prolonged period of controlled viral replication due to ART (median, 86 months), PLWH continue to exhibit alterations in intestinal permeability, persistent inflammatory and immune activation, and disturbances in platelet activation and the coagulation/fibrinolysis system. These factors may synergistically promote atherogenesis.

Previous studies have shown that HIV infection is associated with accelerated progression of carotid atherosclerosis (34). In our study, carotid ultrasound revealed that nearly half of the cohort (49%) had carotid injury, a proportion similar to that reported in a recent Spanish series (35), despite a low estimated 10 years cardiovascular risk according to the REGICOR scale (score of 3). It is important to emphasize that the selected individuals had no clinical signs of cardiovascular or cerebrovascular disease. These findings are striking and consistent with observations in the general population (36), where carotid atherosclerosis is detected in a higher percentage of individuals classified as having a low-moderate cardiovascular risk based on standard risk scales.

A comprehensive assessment was conducted to evaluate potential factors contributing to the increased incidence in PLWH. These included the previously mentioned inflammatory, vascular, platelet and coagulation factors, along with classic cardiovascular risk factors, assessed using two established risk tools: the REGICOR scale (Framingham scale adapted for the Spanish population) (37) and the ASCVD risk estimator (38). Both the REGICOR and ASCVD scores were significantly higher in PLWH with carotid lesions, highlighting the role of classic cardiovascular risk factors, which may be exacerbated by lifestyle habits (39) or ART (40), in this population. Furthermore, PLWH with carotid atherosclerosis showed greater inflammatory activation, as evidenced by elevated serum IL-6 concentrations, compared to those without arterial injury. Multivariable analysis confirmed that both cardiovascular risk factors (as reflected by the REGICOR score) and inflammatory activation (measured by IL-6 levels) were independently associated with the presence of atherosclerotic lesions in PLWH. These findings support a model in which HIV-related chronic inflammation synergizes with conventional risk factors to accelerate atherosclerosis, even in clinically stable patients.

Interleukin-6 is a proinflammatory cytokine released by multiple immune cells, including PMNs, macrophages, lymphocytes, and endothelial cells (41). It is recognized as one of the key initiators of the atherosclerosis process (42), and its levels are known to be increased in PLWH (43). Furthermore, IL-6 has demonstrated prognostic value for overall mortality in PLWH (16, 44–46). Notably, studies have shown that IL-6 is an independent predictor of cardiovascular disease in PLWH, regardless of traditional atherosclerotic risk factors (47, 48). In the REPRIEVE (Reinforced Vascular Event Prevention in HIV) randomized trial, coronary artery disease detected by computed tomography was independently associated with IL-6 levels in PLWH (46). However, the relative contribution of endothelial, platelet, or coagulation factors was not assessed in any of these studies.

In summary, our analysis revealed that, in addition to classical cardiovascular risk factors, IL-6 is independently associated with the presence of subclinical atherosclerosis in PLWH with long-term controlled viral replication, underscoring the relevance of IL-6 as both a marker and a potential therapeutic target in the management of HIV-associated cardiovascular disease. In contrast, other HIV-related factors, such as duration of infection or CD4+ T lymphocyte counts at diagnosis or inclusion, were not identified as independent contributors to the atherosclerotic process. Therefore, our study reinforces the need to integrate inflammatory biomarkers into cardiovascular risk assessment for PLWH, moving beyond traditional risk scores to better identify those at risk of vascular disease. Since current clinical guidelines are mostly based on the general population, there is an urgent need for strategies specifically targeting HIV-related inflammation.

### Limitations

Cardiovascular risk factors were defined according to the guidelines in place at the start of the study (19). Smoking was defined as the consumption of more than 10 cigarettes/day. This criterion differs from more recent definitions that consider any tobacco use. Additionally, the observational and single-center nature of the study may limit the generalizability of the results. Nevertheless, the homogeneity of our cohort and the comprehensive analysis of immuno-inflammatory and vascular markers strengthen the relevance of these findings for PLWH with durable viral suppression.

In conclusion, this study identified significant activation of inflammatory and immune cells, including PMNs, in PLWH with prolonged viral suppression. This immune activation was associated with subclinical atherosclerosis, highlighting its potential role in cardiovascular risk in this population. Additionally, evidence of platelet and coagulation activation was observed. Importantly, the presence of subclinical atherosclerosis was independently influenced by both traditional cardiovascular risk factors and inflammatory activation. These findings support the integration of inflammatory biomarker analysis into the clinical evaluation of PLWH to enable earlier detection and management of atherosclerosis.

## Data Availability

The original contributions presented in this study are included in this article/Supplementary material, further inquiries can be directed to the corresponding author.
