# Supplementary figures and images for: Persistent inflammatory activation in people living with HIV. Involvement in atherosclerosis
Source: Front Med (Lausanne). 2025 Jul 11;12:1621765. doi: 10.3389/fmed.2025.1621765 (PMC12289632; doi:10.3389/fmed.2025.1621765)

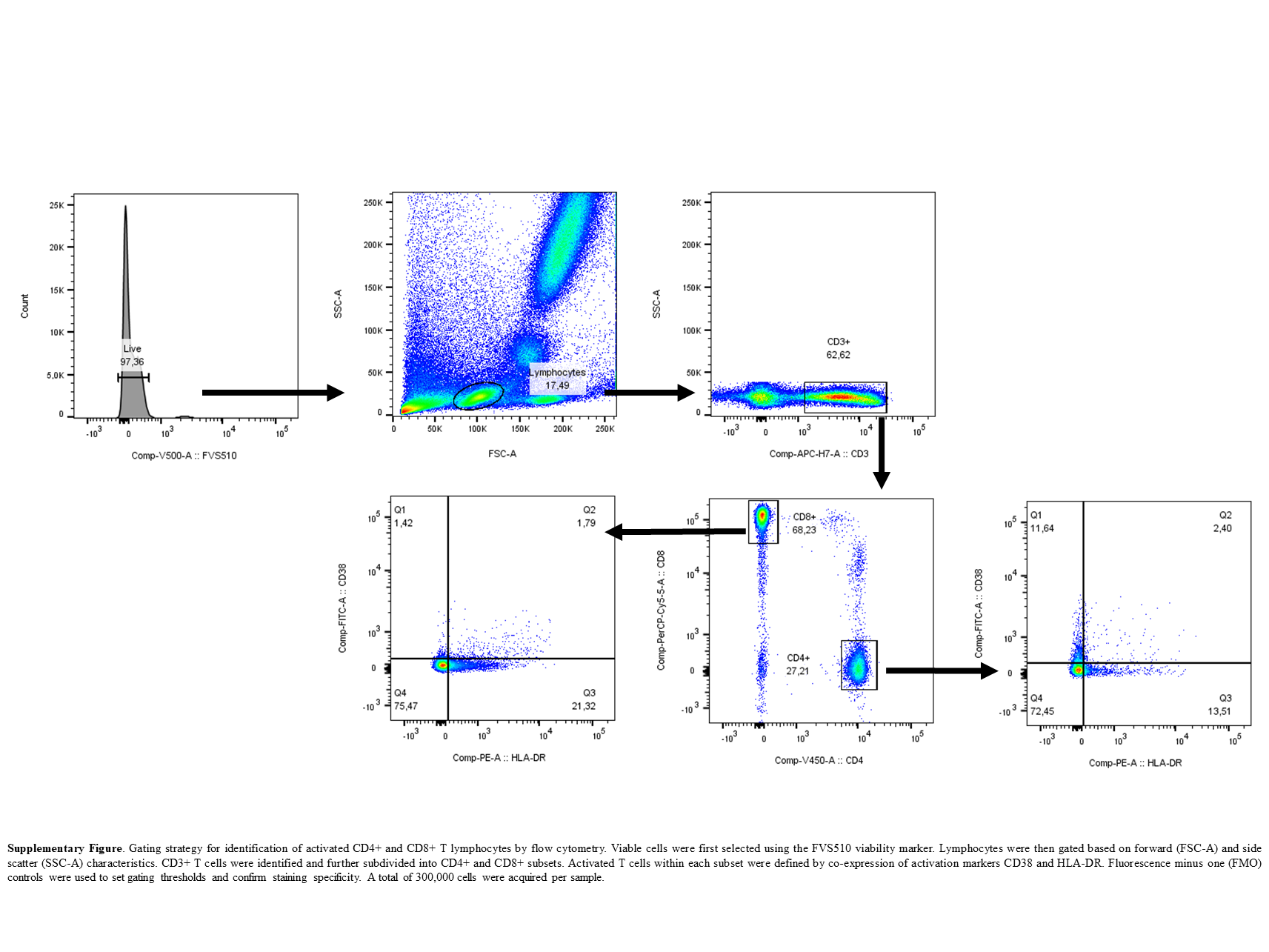

Supplement: Supplementary file 1 [file Image_1.tif]
